# Defects in death-inducing signalling complex formation prevent JNK activation and Fas-mediated apoptosis in DU 145 prostate carcinoma cells

**DOI:** 10.1038/sj.bjc.6601393

**Published:** 2003-11-11

**Authors:** J F Curtin, T G Cotter

**Affiliations:** 1 Tumour Biology Lab, Department of Biochemistry, University College Cork, Lee Maltings, Prospect Row, Cork, Ireland

**Keywords:** CD95, apoptosis, FADD, DAXX, HSP27, JNK

## Abstract

Androgen-independent prostate carcinomas are resistant to chemotherapy and cell lines derived from androgen-independent prostate carcinomas such as DU 145 cells are highly resistant to Fas-mediated apoptosis. The incubation of DU 145 cells with anti-Fas IgM agonistic antibody of Fas receptor fails to activate JNK, a stress kinase involved in regulating apoptosis. We have previously shown that JNK activation is sufficient and necessary to promote Fas-mediated apoptosis in DU 145 cells. We investigate the mechanisms by which JNK activation and apoptosis are abrogated. HSP27 is overexpressed in DU 145 cells and has previously been reported to sequester DAXX and prevent JNK activation in cells treated with anti-Fas IgM. However, we find no evidence that HSP27 interacts with DAXX in DU 145 cells. Instead, we find that FADD does not interact with caspase-8 and this results in defective death-inducing signalling complex formation following Fas receptor activation.

Prostate cancer is the second most common malignancy in the EU, with about 200 000 new cases diagnosed each year. It is a disease that affects primarily elderly men and is responsible for over 35 000 deaths each year (Parkin *et al*, 2001). Androgen-dependent prostate cancer is generally a slowly progressing tumour and treatment with androgen ablation therapy results in tumour regression and an improvement in the quality of life for most patients. However, androgen refractory tumours subsequently develop with a median asymptomatic period of 18 months following androgen ablation (Petrylak, 1999). Androgen-independent tumours are generally more aggressive than androgen-dependent tumours and chemotherapy is only used as a palliative agent (Sternberg, 2001). No single or combined chemotherapeutic regimen has been shown to enhance long-term survival significantly in patients presenting with invasive, hormone refractory prostate cancer (Petrylak, 1999).

The Fas apoptotic pathway has been extensively studied in a variety of tissues and cell types. Tumour cells often develop resistance to Fas receptor-mediated apoptosis as a defence mechanism against the immune system and also against conventional chemotherapeutic agents (Micheau *et al*, 1999; O'Connell *et al*, 2001). Engagement of Fas receptor with Fas ligand or Fas-activating antibodies causes recruitment of procaspase-8 to the death-inducing signalling complex (DISC) through the adapter protein FADD in cell lines sensitive to Fas receptor-mediated apoptosis. Autocleavage and activation of caspase-8 occurs in the DISC and this in turn cleaves a variety of cellular targets, culminating in caspase-3 cleavage and apoptosis (Boldin *et al*, 1996; Muzio *et al*, 1996).

Engagement of Fas receptor with Fas ligand also results in the recruitment of a variety of proteins not directly involved in caspase-8 recruitment and cleavage. These proteins are responsible for initiating other signal transduction pathways from Fas receptor. One protein recruited to the DISC following Fas receptor activation is DAXX, normally present in the nucleus of cells. DAXX binds to Fas receptor at a different site to FADD and is responsible for the recruitment and activation of the kinase ASK1. ASK1 in turn activates the MAPK cascade resulting in JNK activation (Chang *et al*, 1998; Tobiume *et al*, 2001). JNK can also be activated by a caspase-8-dependent mechanism involving cleavage of Mst1 (Graves *et al*, 2001) or MEKK1 (Deak *et al*, 1998), and JNK activation has been reported to enhance Fas receptor-mediated apoptosis in some cell lines (Brenner *et al*, 1997; Yang *et al*, 1997; Le-Niculescu *et al*, 1999; Costa-Pereira *et al*, 2000; Zhang *et al*, 2000).

Upregulation of heat-shock transcription factor-1 was reported to occur in metastatic prostate cancer cell lines. This results in increased expression of HSP27 (Hoang *et al*, 2000), and is invariably associated with poor clinical outcome in patients with advanced prostate cancer (Cornford *et al*, 2000). HSP27 can protect cells from a variety of apoptotic insults including Fas-mediated apoptosis and various chemotherapeutic drugs (Mehlen *et al*, 1996; Samali and Cotter, 1996) by sequestering cytochrome *c* after it is released from the mitochondria and preventing caspase-9 activation (Garrido *et al*, 1999). It can also prevent cytochrome *c* release by inhibiting Bid translocation to the mitochondrion (Paul *et al*, 2002). HSP27 can bind to and prevent the translocation of DAXX to the plasma membrane following Fas receptor activation, thus inhibiting JNK activation and the proapoptotic function associated with DAXX (Charette *et al*, 2000).

DU 145 prostate carcinoma cell lines are highly resistant to Fas-mediated apoptosis. This resistance can be overcome by coadministering sublethal concentrations of various chemotherapeutic drugs (Rokhlin *et al*, 1997; Costa-Pereira and Cotter, 1999). Our group has previously demonstrated that JNK activation is sufficient to sensitise DU 145 cells to Fas receptor-mediated apoptosis (Costa-Pereira *et al*, 2000; Curtin and Cotter, 2002). However, we found that engagement of Fas receptor with Fas-activating antibodies could not activate JNK in DU 145 cells. In order to understand the mechanism by which DU 145 cells are resistant to Fas, we explored the events inhibiting JNK activation. We found that DAXX did not translocate from the nucleus to the cytoplasm following stimulation of Fas. Although HSP27 was highly overexpressed, it did not appear to play a role in this process by sequestering DAXX. Procaspase-8 was not cleaved following Fas receptor activation and further investigation demonstrated that defective DISC formation was the underlying cause by which Fas receptor activation failed to activate either JNK or caspase-8.

## MATERIALS AND METHODS

### 

#### Cell lines and reagents

DU 145 and Jurkat T cells were obtained from American Type Culture Collection (ATCC, Rockville, MD, USA). Cell culture reagents were purchased from Sigma (UK). The fluorescent probes used to detect apoptosis were FITC-conjugated Annexin V (IQ Products, The Netherlands) and propidium iodide (PI) (Sigma, UK). SB203580 and z-VAD-fmk were purchased from Calbiochem (UK). The antibodies used in this study were Fas-activating mouse anti-Fas IgM clone CH-11 and rabbit anti-FADD (Upstate Biotechnology, UK), mouse anti-Fas IgG (Bender Med Systems, Silverstone, Towcester, UK), phospho-JNK (Thr183/Tyr185) clone G9 and mouse anti-caspase-8 (Cell Signalling Technology, UK), rabbit anti-JNK1 and rabbit anti-DAXX (Santa Cruz, USA), mouse anti-PARP (PharMingen, BD Biosciences, UK), mouse anti-HSP27 (Stressgen, UK) and mouse anti-actin (Sigma, UK). The HRP-labelled anti-rabbit IgG and anti-mouse IgG antibodies were obtained from DAKO (Denmark), while FITC-conjugated anti-rabbit IgG was purchased from Sigma (UK). Protein G–agarose slurry was obtained from Peirce (UK) and all other reagents were obtained from Sigma (UK). Anti-Fas IgM was stored at 10 *μ*g ml^−1^ in PBS at −20°C, SB203580 was stored at 5 mM in DMSO at −20°C and z-VAD-fmk was stored at 25 mM in DMSO at −20°C.

### Cell culture

DU 145 cells were cultured in RPMI 1640 medium supplemented with 5% FCS, 2 mM L-glutamine and 10 IU ml^−1^ penicillin–streptomycin. Jurkat cells were cultured in RPMI 1640 medium containing 10% FCS, 2 mM L-glutamine and 10 IU ml^−1^ penicillin–streptomycin. Cells were cultured at 37°C in a humidified atmosphere with 5% CO_2_ and were routinely subcultured every 2–3 days. DU 145 cells were grown to 75% confluency and Jurkat cells were resuspended in fresh media at 0.5 × 10^6^ ml^−1^ on the day of each experiment. Cells were incubated with 200 ng ml^−1^ anti-Fas IgM for 1, 4 or 24 h and pretreated for 1 h with 25 *μ*M z-VAD-fmk or 5 *μ*M SB203580, as indicated in the figure legends.

### Fas receptor expression

A total of 0.5 × 10^6^ cells were used per sample and were harvested and washed twice in PBS. They were stained for 1 h at 4°C with 20 *μ*g ml^−1^ of mouse anti-Fas IgG (1/50). After another two washes with PBS, the cells were stained with the FITC-conjugated sheep anti-mouse IgG (1/40) for 1 h at 4°C in the dark. Cells stained with secondary antibody alone were used to compensate for intrinsic fluorescence and nonspecific binding of the secondary antibody. The samples were read on a FACScan flow cytometer and the data were analysed using Cell Quest software (Beckton Dickenson, UK).

### Annexin V binding and PI uptake assay

DU 145 and Jurkat cells were incubated with 200 ng ml^−1^ anti-Fas IgM for 4 and 24 h as indicated. The cells were subsequently harvested, washed once in PBS and resuspended in 200 *μ*l Annexin V binding buffer (150 mM NaCl, 18 mM CaCl_2_, 10 mM HEPES, 5 mM KCl, 1 mM MgCl_2_). A measure of 1 *μ*g ml^−1^ FITC-conjugated Annexin V, which binds specifically to external phosphatidyl serine on apoptotic cells, was added to each sample and incubated at room temperature for 5 min. PI (50 *μ*g ml^−1^) was added immediately prior to reading the samples on the FACScan. Viable cells exclude PI and stain negative on FL-2. Apoptotic cells are labelled with Annexin V and stain positive on FL-1. Analysis was carried out using Cell Quest Software.

### SDS–PAGE and Western blot

Protein extracts were prepared from cells using RIPA lysis buffer (50 mM Tris, pH 7.4, 150 mM NaCl, 1 mM each of NaF, NaVO_4_ and EGTA, 1% NP40, 0.25% sodium deoxycholate, 0.2 mM phenylmethylsulphonyl fluoride, 1 *μ*g ml^−1^ each of antipain, aprotinin and chymostatin, 0.1 *μ*g ml^−1^ leupeptin, 4.0 *μ*g ml^−1^ pepstatin) and 30 *μ*g of protein in 20 *μ*l SDS–polyacrylamide gel (PAGE) loading dye was loaded in each lane of a 12% SDS–PAGE. Electrophoresis and Western blotting was subsequently carried out. Non-specific protein binding sites were blocked and the membrane was stained with primary and peroxidase-conjugated secondary antibodies according to the manufacturer's recommended protocol. Labelled protein was detected using ECL (Amarsham, UK).

### Immunofluorescent staining of DAXX

DU 145 cells were seeded on glass coverslips and grown to confluency over 48 h. They were incubated with 200 ng ml^−1^ anti-Fas IgM for 4 h or left untreated. The media were aspirated and the coverslips were washed in PBS. The cells were fixed for 15 min at room temperature in 3% PFA in PBS. They were washed in PBS and incubated for 15 min at room temperature in quenching buffer (50 mM NH_4_CL in PBS). Cells were permeabilised with 0.1% Triton X-100 in PBS for 5 min at room temperature and washed in PBS before incubating with primary antibody (1 : 100 in PBS) with 5% FCS for 1 h at room temperature. The primary antibody was aspirated off and the cells were washed in PBS before incubating for 1 h at room temperature in FITC-conjugated secondary antibody (1 : 80 in PBS) with DAPI and 5% FCS. Cells were then washed in PBS and mounted on glass slides.

### Isolation of nuclear- and cytoplasmic-enriched fractions

A minimum of 5 × 10^6^ cells were incubated with 200 ng ml^−1^ anti-Fas IgM for 4 h. The cells were harvested and resuspended in 250 *μ*l homogenising buffer (210 mM mannitol, 70 mM sucrose, 5 mM HEPES, 1 mM EGTA, 0.5% BSA, 1 mM DTT, 0.2 mM PMSF, 5 *μ*g ml^−1^ each of antipain, aprotinin and chymostatin, 0.5 *μ*g ml^−1^ leupeptin, 20 *μ*g ml^−1^ pepstatin). The sample was then transferred to a 2 ml tissue grinding tube (Kontes Glass Company, NJ, USA) and homogenised with 100 strokes of the low clearance pestle. The homogenate was centrifuged at 3000 **g** for 5 min. The supernatant (cytoplasmic fraction) was washed three times at 3000 **g**. The pellet (nuclear fraction) was washed three times in PBS, and the protein extract was prepared using RIPA lysis buffer. SDS–PAGE and Western blotting were performed as described previously.

### Immunoprecipitation

A minimum of 500 *μ*g of protein was used per sample. DU 145 cells were treated and harvested as described in the figure legends. The cells were gently lysed on ice in lysis buffer (10 mM Tris pH 7.5, 50 mM NaCl, 10 mM sodium pyrophosphate, 50 mM NaF, 1 mM NaVO_4_, 1% NP40, 0.2 mM PMSF, 5 *μ*g ml^−1^ each of antipain, aprotinin and chymostatin, 0.5 *μ*g ml^−1^ leupeptin, 20 *μ*g ml^−1^ pepstatin) and centrifuged at 20 000 **g** for 15 min to remove insoluble material. The total cell protein was quantitated and diluted to 1 *μ*g ml^−1^ in PBS. Protease inhibitors were added (1 *μ*g ml^−1^ each of antipain, aprotinin and chymostatin, 0.1 *μ*g ml^−1^ leupeptin, 4 *μ*g ml^−1^ pepstatin), and the samples were incubated with 10 *μ*g ml^−1^ rabbit anti-DAXX or 10 *μ*g ml^−1^ rabbit anti-FADD overnight at 4°C. Protein G–agarose-conjugated beads were prepared according to the manufacturer's recommended instructions and incubated with the samples for a further 1 h at 4°C. DAXX was immunoprecipitated from total protein by centrifugation at 1000 **g** for 3 min. The beads were washed four times in PBS and boiled in SDS–PAGE loading buffer for 5 min. The agarose beads were precipitated out of solution by centrifugation at 20 000 **g** for 2 min and the supernatant was loaded onto an SDS–PAGE and analysed by Western blot as described previously.

## RESULTS

### DU 145 prostate carcinoma cells are resistant to Fas-mediated apoptosis

**Figure 1:**
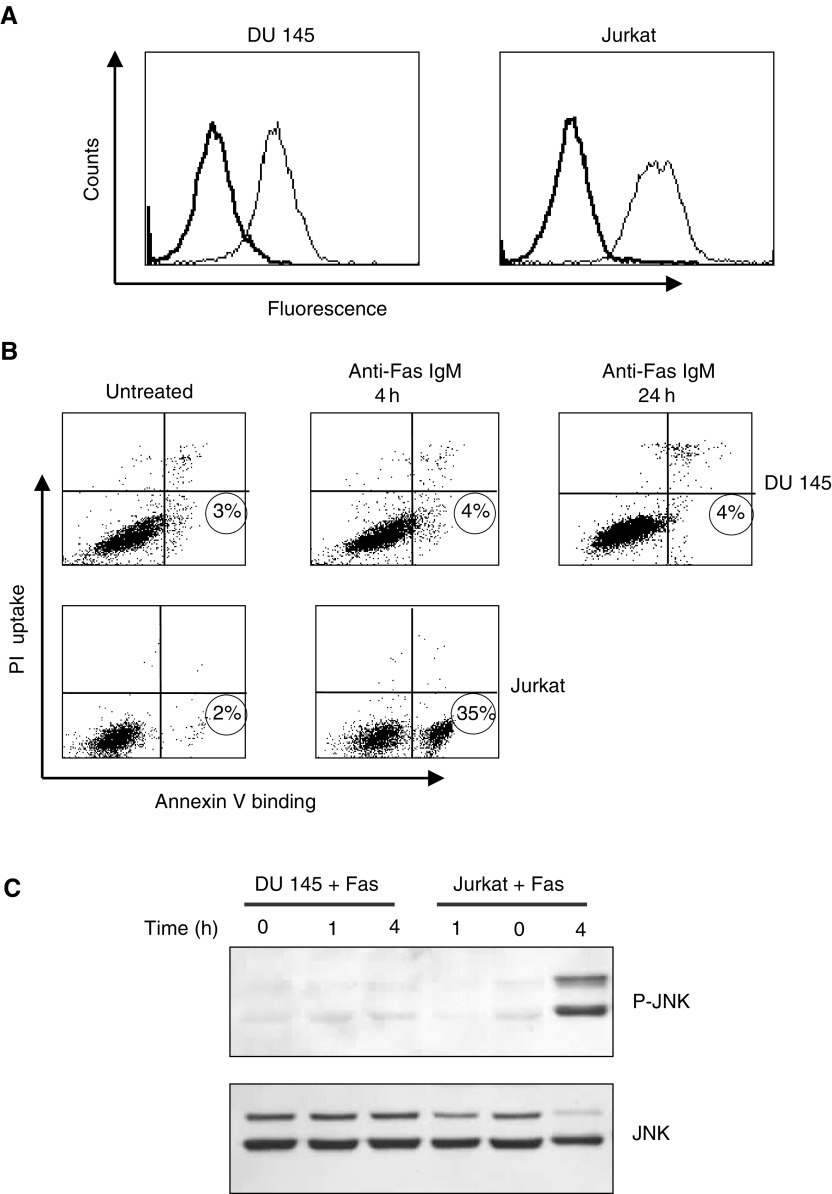
DU 145 cells express Fas receptor at the plasma membrane but are highly resistant to Fas-induced apoptosis. (**A**) Cell surface expression of Fas receptor (**–**) was assessed in DU 145 cells and Jurkat cells as described in the Materials and Methods section. Intrinsic fluorescence of cells labelled with secondary antibody alone (**—**) is also shown. Data are representative of three independent experiments. (**B**) Jurkat cells were incubated for 4 h and DU 145 cells were incubated for 4 and 24 h with 200 ng ml^−1^ anti-Fas IgM. Apoptosis was subsequently determined by staining with Annexin V–FITC and PI. The percentage of apoptotic cells is shown in the bottom right quadrant of each plot. Data are representative of three independent experiments. (**C**) Phosphorylation of JNK at residues Thr 183 and Tyr 185 was assessed in DU 145 cells and Jurkat cells following treatment with 200 ng ml^−1^ anti-Fas IgM for 1 and 4 h where indicated. The total JNK expression was also determined to demonstrate equal protein loading.

Flow cytometry was used to determine the expression of cell surface Fas receptor in DU 145 cells and Jurkat T cells. We found that the expression of Fas receptor was comparable between the two cell lines (Figure 1A

). However, the sensitivity of these two cell lines to Fas-mediated apoptosis was found to be markedly different. Using Annexin V–FITC to detect cells at early stages in apoptosis, Jurkat cells were found to undergo extensive apoptosis in less than 4 h following incubation with 200 ng ml^−1^ anti-Fas IgM. By contrast, no increase in apoptosis was observed in DU 145 cells treated with 200 ng ml^−1^ anti-Fas IgM even for 24 h (Figure 1B). Morphological assessment of apoptosis was used to confirm this observation (data not shown). JNK activation has been reported to accompany Fas receptor activation. Some studies found that JNK was not required for Fas receptor-mediated apoptosis (Abreu-Martin *et al*, 1999; Low *et al*, 1999; Hofmann *et al*, 2001), but others have shown that JNK activation accelerates Fas-mediated apoptosis in a number of cell lines (Brenner *et al*, 1997; Yang *et al*, 1997; Le-Niculescu *et al*, 1999; Zhang *et al*, 2000). Our group has previously identified JNK activation as being necessary for Fas-mediated apoptosis in DU 145 cells (Costa-Pereira *et al*, 2000; Curtin and Cotter, 2002). As a result, we determined the extent of JNK activation in DU 145 cells and Jurkat cells following incubation with 200 ng ml^−1^ anti-Fas IgM for 1 and 4 h. We found that JNK was only extensively phosphorylated in Jurkat cells treated for 4 h with anti-Fas IgM (Figure 1C). No increase in JNK phosphorylation was observed in DU 145 cells even after 24 h (data not shown).

### DAXX is expressed in the nucleus of DU 145 cells

**Figure 2:**
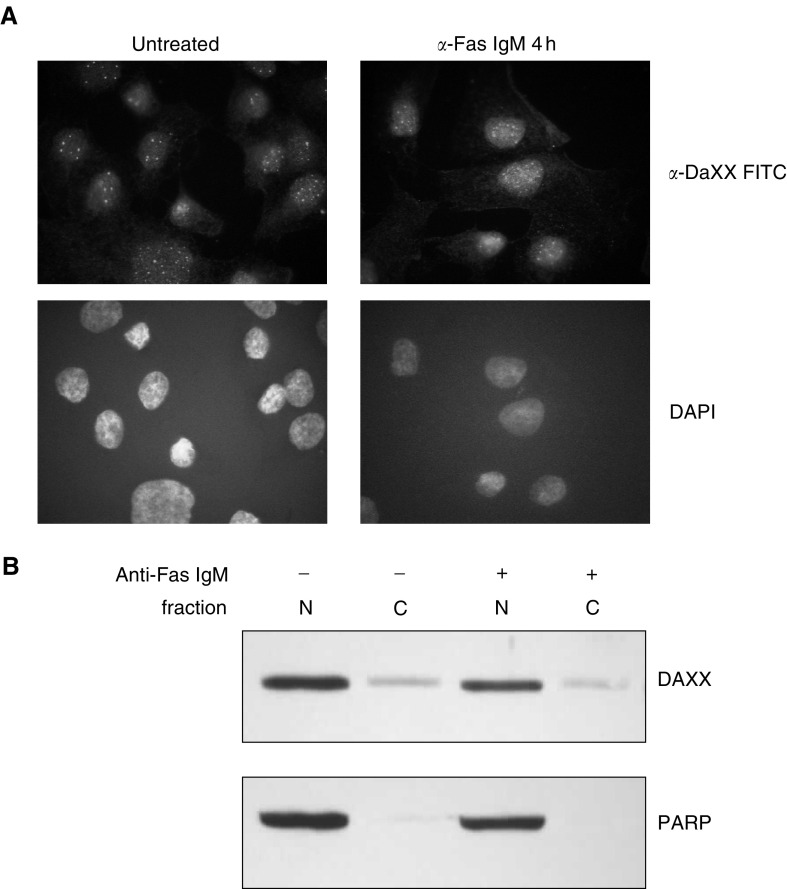
DAXX expression and subcellular localisation in DU 145 cells. (**A**) Immunofluorescent analysis of DAXX was performed on DU 145 cells incubated for 4 h with 200 ng ml^−1^ anti-Fas IgM. Samples were counterstained with DAPI to visualise the nuclei of cells. (**B**) Western blot analysis of DAXX expression in nuclear- and cytoplasmic-enriched fractions obtained from DU 145 cells incubated for 4 h with 200 ng ml^−1^ anti-Fas IgM or without. PARP was also stained to assess the purity of the fractions.

Fas receptor is believed to activate JNK by caspase-dependent and -independent mechanisms. The activation of Fas receptor can recruit DAXX, a nuclear protein, to the plasma membrane during DISC formation. DAXX subsequently binds to and activates ASK1, an upstream kinase in the JNK signalling pathway (Chang *et al*, 1998; Tobiume *et al*, 2001). Immunofluorescence was used to determine DAXX subcellular localisation in DU 145 cells. We found that DAXX was predominately located in the nucleus of DU 145 cells and the staining pattern appeared to be punctated. This is in agreement with other reports that localised DAXX to ND-10 domains in the nucleus (Torii *et al*, 1999; Charette *et al*, 2000). No change in DAXX subcellular localisation was observed following incubation for 4 h with 200 ng ml^−1^ anti-Fas IgM (Figure 2A

). The expression of DAXX was also determined in nuclear- and cytoplasmic-enriched fractions by Western blot. No increase in cytoplasmic DAXX was identified in cells incubated with 200 ng ml^−1^ anti-Fas IgM for 4 h, confirming the immunofluorescence data (Figure 2B). We also assessed the extent of DAXX translocation after 8 and 24 h incubation with anti-Fas IgM and did not observe any increase in the cytoplasmic fraction of DAXX (data not shown).

### HSP27 is overexpressed in DU 145 cells but does not interact with DAXX

**Figure 3:**
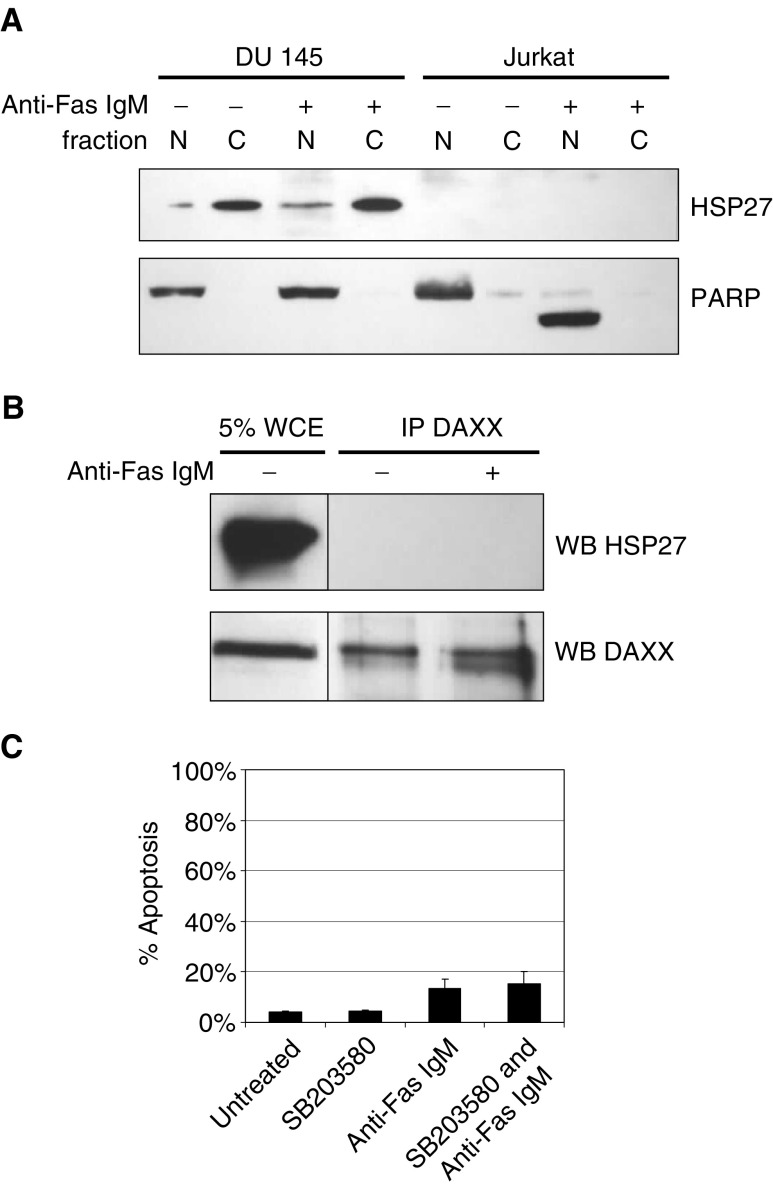
HSP27 is overexpressed in DU 145 cells, but is localised predominantly in the cytoplasm and does not coimmunoprecipitate with DAXX. (**A**) Expression of HSP27 was analysed in nuclear- and cytoplasmic-enriched fractions isolated from DU 145 cells and Jurkat cells before and after incubation with 200 ng ml^−1^ anti-Fas IgM for 4 h. PARP was also probed to determine the purity of the fractions. (**B**) DU 145 cells were treated for 4 h with 200 ng ml^−1^ anti-Fas IgM or left untreated. Cells were lysed gently and DAXX was immunoprecipitated as described in the Materials and Methods section. Immunoprecipitated complexes and 5% whole-cell extract from untreated DU 145 cells were subsequently analysed for HSP27 expression by Western blot. DAXX was probed to determine equal protein loading. (**C**) DU 145 cells were stained with Annexin V–FITC and PI to determine the extent of apoptosis following incubation for 24 h with 5 *μ*M SB203580 and 200 ng ml^−1^ anti-Fas IgM. Error bars represent the standard deviation from the mean for three independent experiments.

HSP27 has previously been found to bind and inhibit DAXX translocation and apoptosis in response to Fas receptor activation (Charette *et al*, 2000). In addition, the overexpression of HSP27 correlates with prostate cancer progression (Cornford *et al*, 2000). Therefore, we analysed whether HSP27 inhibited JNK activation following Fas receptor activation in DU 145 cells. HSP27 expression was determined in nuclear- and cytoplasmic-enriched fractions from DU 145 cells and Jurkat cells. We found that HSP27 was highly overexpressed in DU 145 cells and was predominantly located in cytoplasmic-enriched fractions. No change in subcellular expression was observed following treatment with 200 ng ml^−1^ anti-Fas IgM for 4 h (Figure 3A

). Although HSP27 is expressed mainly in the cytoplasm and DAXX is present primarily in the nucleus, it is possible that the cytoplasmic DAXX is important for binding Fas receptor or that HSP27 prevents recruitment of nuclear DAXX to the Fas DISC. As a result, we immunoprecipitated DAXX from cell lysates of DU 145 cells incubated for 4 h with 200 ng ml^−1^ anti-Fas IgM or left untreated. We could not detect any HSP27 expressed in the immunoprecipitate, suggesting that either HSP27 and DAXX do not interact in DU 145 cells or the percentage HSP27 that interacts with DAXX is extremely small in comparison with total HSP27 expression in these cells (Figure 3B). It was reported that endogenous P38 activity maintained HSP27 in active dimers. The inhibition of P38 resulted in multimeric complexes of HSP27 and this abrogated the interaction between HSP27 and DAXX. In addition, SB203580 sensitised cells to Fas receptor-mediated apoptosis by allowing DAXX translocation from the nucleus to the cytoplasm (Charette *et al*, 2000). We did not observe any increase in cytoplasmic DAXX (data not shown) or increase in apoptosis in cells preincubated with SB203580 (Figure 3C). This supports our conclusion that HSP27 does not prevent DAXX translocation and JNK activation in DU 145 cells.

### Fas-mediated caspase activation is required for JNK activation in Jurkat cells but is defective in DU 145 cells

**Figure 4:**
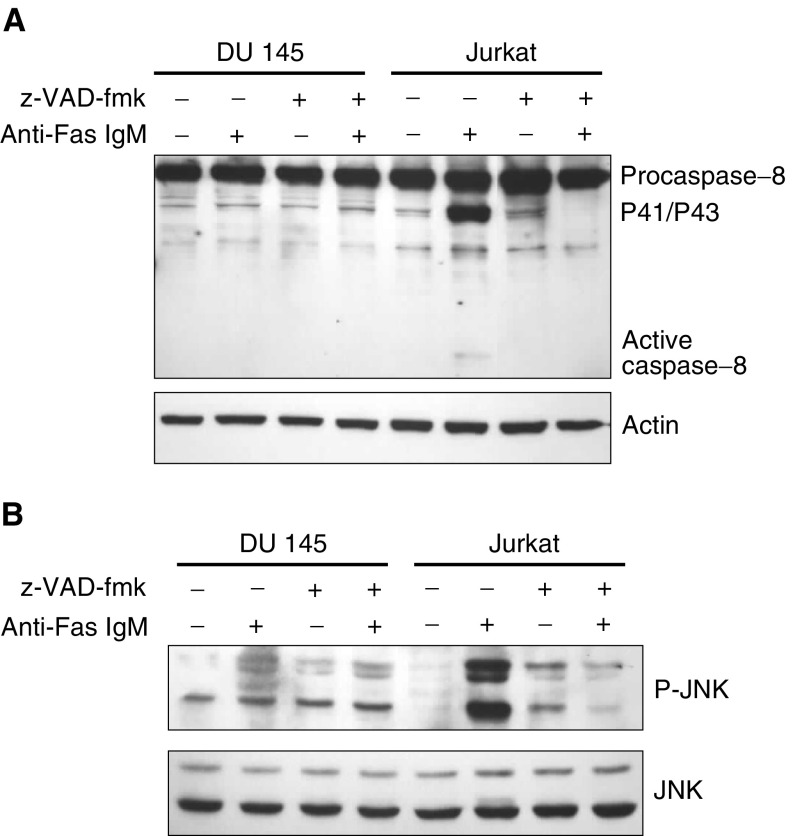
Inhibition of caspase activity with z-VAD-fmk completely abrogates JNK activation in Jurkat cells. (**A**) DU 145 cells and Jurkat cells were incubated with 25 *μ*M z-VAD-fmk and 200 ng ml^−1^ anti-Fas IgM for 4 h as outlined above. Cell lysates were subsequently probed for caspase-8 expression and cleavage. Cleavage of procaspase-8 into intermediary P41/P43 and active P18 caspase-8 subunits was only evident in Jurkat cells incubated with 200 ng ml^−1^ anti-Fas IgM alone. Actin was also probed to assess equal protein loading. (**B**) DU 145 cells and Jurkat cells were incubated with 25 *μ*M z-VAD-fmk and 200 ng ml^−1^ anti-Fas IgM for 4 h. Phosphorylation of JNK was assessed in cell lysates by Western blot. The total JNK expression was also assessed to demonstrate equal protein loading.

In order to study caspase-dependent JNK activation, we incubated DU 145 and Jurkat cells with 200 ng ml^−1^ anti-Fas IgM in the presence and absence of the general caspase inhibitor z-VAD-fmk for 4 h. Cleavage of procaspase-8 was subsequently assessed by Western blot analysis. We found that procaspase-8 was extensively cleaved into the intermediate P41/P43 products and active P18 subunit only in Jurkat cells following incubation with 200 ng ml^−1^ anti-Fas IgM. Z-VAD-fmk was found to abrogate the cleavage of caspase-8 completely. Procaspase-8 was not cleaved following treatment of DU 145 cells with 200 ng ml^−1^ anti-Fas IgM. This indicates that inhibition of Fas-mediated apoptosis is an early event in these cells, possibly during DISC formation (Figure 4A

). The effect of caspase inhibition on JNK activation was also assessed in Jurkat cells. Z-VAD-fmk was found to abrogate JNK activation completely in Jurkat cells following incubation with 200 ng ml^−1^ anti-Fas IgM. This suggests that in this cell line, the principle mechanism of JNK activation is caspase dependent (Figure 4B).

### DISC formation following Fas receptor activation is defective in DU 145 cells

**Figure 5:**
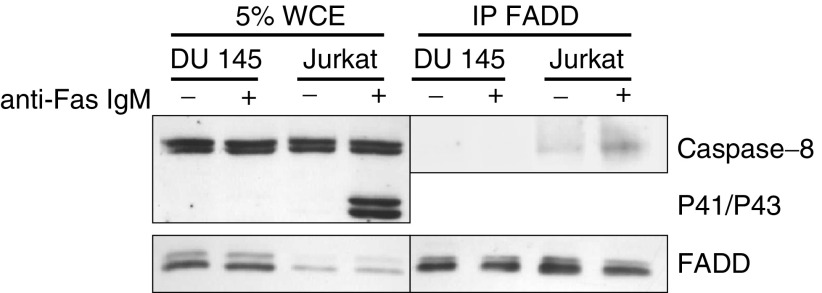
Interaction between FADD and caspase-8 is defective in DU 145 cells. FADD was immunoprecipitated from DU 145 and Jurkat cells before and after incubation with 200 ng ml^−1^ anti-Fas IgM. The samples were probed for caspase-8 and FADD expression by Western blot. The endogenous expression of FADD and caspase-8 in 5% whole-cell extracts was also determined.

Although cell surface Fas receptor expression is similar in DU 145 and Jurkat cells, we found that neither JNK nor caspase-8 are activated in DU 145 cells. This may be due to defective DISC formation following Fas receptor activation in DU 145 cells. We immunoprecipitated FADD from cells before and after incubation with 200 ng ml^−1^ anti-Fas IgM for 1 h. The expression of caspase-8 was assessed in these immunoprecipitates to determine the extent of FADD-caspase-8 aggregation in the DISC. While FADD was found to associate with caspase-8 in Jurkat cells incubated with anti-Fas IgM, no interaction was evident in DU 145 cells (Figure 5

). These data suggest that the interaction between caspase-8 and FADD is defective and prevents DISC formation following Fas receptor activation in DU 145 cells. This appears to be the principle mechanism by which DU 145 cells are resistant to anti-Fas IgM, and also why JNK is not activated in these cells.

## DISCUSSION

The activation of the Fas receptor in cells by Fas ligand results in caspase activation and morphological apoptosis in a variety of cell lines. Fas receptor plays a critical role in the homeostasis of the immune system and may be involved in immune surveillance and clearance of defective cells (Pinkoski and Green, 2000; O'Connell *et al*, 2001). Although the events initiated by Fas receptor culminating in caspase activation and apoptosis are well understood, the mechanisms by which tumour cells alter signalling pathways and become resistant to Fas-mediated apoptosis are not. DU 145 cells are androgen-independent prostate carcinoma cells and are resistant to a variety of chemotherapeutic drugs *in vitro*. We found that while cell surface expression of Fas receptor was comparable to Jurkat cells, DU 145 cells were highly resistant to Fas-mediated apoptosis. The activation of JNK using chemotherapeutic drugs or anisomycin was sufficient to sensitise these cells to Fas (Costa-Pereira and Cotter, 1999; Costa-Pereira *et al*, 2000; Curtin and Cotter, 2002).

JNK activation has been reported to accompany Fas receptor activation and appears to be involved in regulating Fas-mediated apoptosis in various cell lines. While JNK is not proapoptotic in every cell line, it appears that certain cell lines resistant to Fas-mediated apoptosis require JNK activation to promote apoptosis. We found that the treatment of DU 145 cells with anti-Fas IgM alone did not stimulate JNK activation. In order to better understand the resistance of DU 145 cells to Fas-mediated apoptosis, we investigated JNK activation following Fas receptor stimulation in DU 145 cells. JNK activation following Fas receptor activation may be caspase-8 dependent or caspase-8 independent. During caspase-8-independent JNK activation, DAXX is recruited to the plasma membrane and binds to the intracellular C-terminus of Fas receptor independent of FADD. ASK1, a JNK kinase kinase is recruited to the plasma membrane and binds to DAXX. The activation of ASK1 *in trans* results in MKK4/JNKK1 phosphorylation and ultimately JNK phosphorylation at Thr183/Tyr185 and activation (Tobiume *et al*, 2001). DU 145 cells were found to express DAXX predominantly in ND-10 domains in the nucleus and this is consistent with previous reports (Torii *et al*, 1999; Charette *et al*, 2000). We found that a small fraction of DAXX was present in the cytoplasmic fraction, although the levels of cytoplasmic DAXX were not found to increase following Fas receptor activation. In addition, no clustering of DAXX at the plasma membrane was evident in cells treated with anti-Fas IgM.

HSP27 overexpression has been associated with prostate cancer progression and can independently predict the clinical outcome of prostate cancer, suggesting that it plays an important role in the resistance of prostate cancer to chemotherapy (Thomas *et al*, 1996; Cornford *et al*, 2000). HSP27 inhibits apoptosis by a variety of mechanisms, including sequestering cytosolic proapoptotic Cytochrome *c*, inhibiting proapoptotic tBID translocation from cytosol to the mitochondrion and preventing DAXX association with Fas receptor and subsequent JNK activation (Concannon *et al*, 2003). We found that DU 145 cells overexpress HSP27 and is predominantly found in the cytoplasmic fraction. A small fraction present in the nucleus was also evident. However, we could not identify any physical interaction between HSP27 and DAXX in DU 145 cells either before or after Fas receptor activation. This suggests that it is not involved in regulating DISC formation and JNK activation in DU 145 cells. It is likely that HSP27 regulates the sensitivity of mitochondria to apoptosis signals and can prevent cytochrome *c* release in response to cytotoxic drugs because HSP27 overexpression correlates with poor clinical outcome (Cornford *et al*, 2000). However, another mechanism inhibits Fas receptor-mediated apoptosis and JNK activation in DU 145 cells.

Caspase-8 activation results in the cleavage and constitutive activation of MEKK1 and Mst1, kinases that can phosphorylate and activate JNK. We found that caspase-8 is expressed at similar levels in DU 145 cells and Jurkat cells, but Fas receptor engagement with Fas-activating antibodies was found only to cleave procaspase-8 into active fragments in Jurkat cells. This cleavage could be completely abrogated using z-VAD-fmk, an irreversible caspase inhibitor. JNK phosphorylation was also completely inhibited in cells lacking caspase-8 active fragments, suggesting that caspase-8-mediated JNK activation was the predominant pathway in Jurkat cells, at least after 4 h. It is possible that caspase-8-independent JNK activation can also occur here, but progresses more slowly.

In light of our data, defective DISC formation following Fas receptor activation appeared to be the mechanism by which DU 145 cells were resistant to Fas-mediated apoptosis. We immunoprecipitated FADD, the adaptor protein required for caspase-8 recruitment to Fas receptor, to determine the extent of interactions between FADD and caspase-8 in DU 145 cells and Jurkat cells before and after Fas stimulation. Although DU 145 cells appear to express higher levels of FADD than Jurkat cells, no interaction between FADD and caspase-8 was evident before or after Fas receptor stimulation. By contrast, caspase-8 was found to immunoprecipitate with FADD in both untreated and anti-Fas IgM-treated Jurkat cells. Increased caspase-8 in anti-Fas IgM-treated Jurkat cell immunoprecipitates was consistently observed and this is probably due to stable interactions between FADD and caspase-8 in Fas receptor aggregates.

Numerous Fas receptor and FADD interacting proteins have been identified and a number of these have been shown to regulate DISC formation following Fas receptor engagement with Fas ligand and Fas-activating antibodies. These include FAP-1, FAF-1, FLASH, HIPK3 and PKC*ξ* (Peter and Krammer, 2003). It is likely that one or more of these proteins are differentially regulated in prostate cancer and as a result increase the threshold required for Fas receptor activation and apoptosis following engagement of Fas receptor with Fas ligand. Further studies are ongoing with the aim of identifying these components. It is hoped that by identifying the dysfunctional elements in Fas receptor-mediated apoptosis in DU 145 cells novel therapeutic targets may be identified for prostate cancer.
